# Predicting violent infractions in a Swiss state penitentiary: A replication study of the PCL-R in a population of sex and violent offenders

**DOI:** 10.1186/1471-244X-8-74

**Published:** 2008-09-08

**Authors:** Jérôme Endrass, Astrid Rossegger, Frank Urbaniok, Arja Laubacher, Stefan Vetter

**Affiliations:** 1 Psychiatric/Psychological Service, Criminal Justice System, Canton of Zurich, Zurich, Switzerland; 2 Centre for Disaster and Military Psychiatry, University of Zurich, Zurich, Switzerland

## Abstract

**Background:**

Research conducted with forensic psychiatric patients found moderate correlations between violence in institutions and psychopathy. It is unclear though, whether the PCL-R is an accurate instrument for predicting aggressive behavior in prisons. Results seem to indicate that the instrument is better suited for predicting verbal rather than physical aggression of prison inmates.

**Methods:**

PCL-R scores were assessed for a sample of 113 imprisoned sex and violent offenders in Switzerland. Logistic regression analyses were used to estimate physical and verbal aggression as a function of the PCL-R sum score. Additionally, stratified analyses were conducted for Factor 1 and 2. Infractions were analyzed as to their motives and consequences.

**Results:**

The mean score of the PCL-R was 12 points. Neither the relationship between physical aggression and the sum score of the PCL-R, nor the relationship between physical aggression and either of the two factors of the PCL-R were significant. Both the sum score and Factor 1 predicted the occurrence of verbal aggression (AUC = 0.70 and 0.69), while Factor 2 did not.

**Conclusion:**

Possible explanations are discussed for the weak relationship between PCL-R scores and physically aggressive behavior during imprisonment. Some authors have discussed whether the low base rate of violent infractions can be considered an explanation for the non-significant relation between PCL-R-score and violence. The base rate in this study, however, with 27%, was not low. It is proposed that the distinction between reactive and instrumental motives of institutional violence must be considered when examining the usefulness of the PCL-R in predicting in-prison physical aggressive behavior.

## Background

Psychopaths are characterized by specific deficits in interpersonal relations, affective attributes and behavioral features [1-3]. The Psychopathy Checklist-Revised (PCL-R) [2] was designed to measure these attributes and is considered to be the gold standard for the diagnosis of psychopathy, as its diagnostic validity is well replicated [4]. The PCL-R consists of 20 items, which are assessed on a three-step scale. Factor analyses of the PCL-R items found two factors: Factor 1 represents items which assess interpersonal and affective characteristics, and Factor 2 represents behavioral and lifestyle factors, such as impulsivity and antisocial behaviors. Other studies have even found a much debated three [5] and a robust four factor model of psychopathy [6]. Even though the majority of studies examining the predictive validity of the PCL-R for criminal recidivism were conducted on mostly Caucasian North American prisoners, several studies were able to demonstrate the usefulness of this instrument for ethnically diverse populations in English-speaking countries [7,8]. All in all, the PCL-R score can be considered a solid and accurate estimate for recidivism and especially violent recidivism in English-speaking countries, as well as in Europe [7,9,10]. In German-speaking countries, there have been few studies examining the validity of the PCL-R and the PCL:SV in predicting re-offending and they confirm, at the least, a moderate predictive validity of the instrument [11-13]. A study examining 428 forensically assessed offenders in Switzerland also found a moderate accuracy for the screening version of the PCL-R, the PCL:SV, with an AUC value of 0.65 (mean score in the PCL:SV: 9.4 points) [14].

For many professionals working in the criminal justice system, the question arises whether the PCL-R is also able to predict violence during institutionalization (correctional facility, forensic hospital, psychiatric hospital). The relationship between psychopathy and inpatient disruptive behavior has been studied in numerous samples of – mostly North American – male prisoners and psychiatric patients [15]. Early studies reported a significant association [16,17], but their methodology was criticized by Cunningham and Reidy [18] on the grounds that the criterion of interest had not been adequately defined. More recent studies among prison populations indicated only a moderate association between psychopathy and inmate misbehavior [19-22]. More specifically, research conducted with *forensic psychiatric patients *found at least a moderate correlation between violence in institutions and psychopathy [15,20,23,24]. Rice, Harris and Cormier [25] compared high and low PCL-R scores in a Canadian forensic hospital and found an association between high PCL-R scores (PCL-R > 25) and a higher level of behavioral problems during treatment, including more episodes of seclusion during the first and last year of treatment. Gray, Hill, McGleish, Timmons, MacCulloch, and Snowden, [26] studied 34 mentally disordered offenders from a medium-security hospital in the U.K. The total PCL-R score had moderate predictive validity for damage to property and physical violence. In a sample of 218 male offenders (aged 17–71 years) admitted to a State Hospital with psychiatric disorders, Heilbrun et al. [20] found a significant correlation between the total number of aggressive incidents in the first 2 months of hospitalization and the total PCL score. Douglas et al. [4] found a mean score of 13 for the Psychopathy Checklist Screening Version (PCL:SV) among 216 male forensic patients of a Swedish hospital [27]. The AUC of the PCL:SV in predicting aggression was 0.63. On the other hand, there are – interestingly – only few studies examining the relationship between PCL-R score and violent behavior among *prison inmates*. Edens et al. [19] examined the discriminative validity of the PCL-R among an ethnically diverse, young, and randomly selected population of 50 male English-speaking inmates of a US prison. The data showed a generally modest but statistically significant correlation between the PCL-R and indexes of aggressive institutional behavior during the first year of incarceration: The correlation between PCL-R score and physical aggression was 0.18 (not significant) but 0.28 for verbal aggression (significant). Kroner and Mills [28] examined institutional misconduct in 97 violent offenders sentenced to 2 to 6 years in Canada over an 8 month period. The mean PCL-R score was 19.7 and the prevalence of major misconduct was 36%, including e.g. rioting, threatening, drug use and assault, however, only two incidents were concerned with threatening or attempting to assault staff. The authors found that the PCL-R was barely predictive (r = 0.14, AUC = .58). Edens, Buffington-Vollum, Colwell, Johnson and Johnson [29] examined 92 incarcerated sex offenders. They found that the sum score was predictive for physical and verbal aggressive infractions. This finding contrasts somewhat with the results of Buffington-Vollum, Edens, Johnson and Johnson [30] in a prospective study of 58 sex offenders incarcerated in the Texas Department of Criminal Justice. They reported base rates for physical and verbal aggression of 8% and 39%. The authors concluded that the PCL-R correlated moderately with verbal aggression, non-aggressive offenses, and 'any disciplinary offense', but not with physically aggressive offenses. Coid, Petruckevitch, Bebbington, Jenkins, Brugha, Lewis, et al. [31] examined psychopathy in a sample of 496 prisoners in England and Wales using the PCL-R and found that a higher proportion of inmates segregated due to disciplinary infractions scored 25 or higher on the PCL-R (OR = 3.08).

There is some evidence for the usefulness of the PCL-R in predicting disciplinary infractions in institutions. It is unclear, though, whether the PCL-R is an accurate instrument to predict violent behavior in prisons, and results seem to indicate that the instrument is better suited for predicting verbal rather than physical aggression. The objective of this study was to examine the predictive validity of the PCL-R for physical and verbal aggression in a sample of imprisoned sex and violent offenders in Switzerland. In addition, the motives and consequences of the aggressive behavior were analyzed.

## Methods

### Ethical approval

The sample of the present study is a subsample of a large epidemiological study conducted on convicted offenders (inmates as well as offenders on probation) in the Canton of Zurich in the year 2000, which was approved as a whole by an external Ethics Committee, the Kantonale Ethikkommission Zürich (KEK – ). In agreement with the committee, no informed consent had to be obtained as there was no contact with any of the study subjects. All data was collected entirely from the subjects' files and anonymised before further analysis.

### Characteristics of the Swiss penitentiary

The subjects examined in the present study were all inmates of the maximum security unit of the state penitentiary "Pöschwies" in the Canton of Zurich, Switzerland. The "Pöschwies" is the largest and most modern penitentiary in Switzerland and has space for 436 inmates. The penitentiary contains three prisons. The main prison is a *maximum security unit *and contains offenders, who have to serve at least a 2 years prison term. The second prison is a *medium security unit *and contains offenders who serve short prison sentences and the third prison is a *minimum security unit *which contains mostly first time offenders and offenders from the maximum security unit before they are discharged to a halfway house.

In the maximum security unit every prisoner has his own cell and can rent a private television set, as well as a computer. The doors of the cells are locked for approximately 12 hours a day (during the night). The prisoners have to work 7 hours a day, from Monday to Friday, in the industrial workshops of the penitentiary. There are 11 different industrial workshops (e.g. print office, bakery, laundry, painter's shop, joiner's workshop, mechanic's workshop, bookbindery). Depending on their vocational qualification, the prisoners can earn up to 600 Swiss Francs (approximately 550$) per month and have the opportunity to enroll in a vocational education program. Inmates live in group homes, which house up to 20 persons each. All members of a group home have their meals in their own refectory, and have the possibility of spending time together in their own sitting room. Furthermore, in their spare time, the inmates can practice sports, participate in courses or spend time in their cell. Offenders who do not present an acute risk to others are allowed to see visitors once a week. Married offenders can use a private room for conjugal visits. Aside from the regular group homes there are special needs group homes: One for offenders with long-term prison sentences, one for high risk offenders, one for offenders with substance use problems and one group home for older prisoners. The inmates of the special needs group homes benefit from several privileges: Their cells are locked later in the evening, they don't have to go to work, they can cook their own meals etc.

270 prison officers and master-workmen take care of 436 inmates. In case a prisoner needs medical care there are two general practitioners, two psychiatrists, two part time dentists, two part time physical therapists and four nurses available. The inmates can also apply for offense-oriented psychotherapy. There are currently nine fulltime forensic psychologists offering specialized treatment programs. Approximately 15% of the offenders (n = 70) take part in a single and/or group therapy with an intensity of 1 to 12 hours per week.

### Sample selection criteria

Only the inmates of the maximum security unit were examined for inclusion in the study. The inclusion criteria were: (1) conviction due to a violent or sex offense, (2) sentenced to at least 10 months imprisonment, (3) administrated by the Zurich correctional and probation service in August 2000, and (4) existence of a psychiatric expert assessment in the offender's prison records. 123 offenders fulfilled these inclusion criteria. The exclusion of all subjects with more than four omitted items in the PCL-R reduced the sample to 113 subjects.

All subjects were male, 57.5% (n = 65) were Swiss nationals and 12.3% (n = 14) originated from an EU country. Further countries of origin were Russia, China, the Philippines, Sri Lanka, Peru, the Dominican Republic, Jamaica, Croatia, Lebanon, and Turkey. The mean age at the beginning of the sentence was 36.3 years (SD = 8.9, range: 20–60). At the time of the offense, 15.2% (n = 17) of the offenders were married, 34.9% (n = 38) had a child, and 31.2% (n = 34) had lived in a foster home before the age of 15. 83.2% (n = 94) of the offenders had a criminal record prior to the index offense and 31.2% (n = 34) had previously been treated in an inpatient psychiatric facility. Psychiatric disorders according to ICD-10 [32], with 86.7% (n = 98), were very prevalent in our sample. Over half of the 114 offenders had been diagnosed with an affective disorder (56.6%; n = 64) and 16.8% (n = 19) with a personality disorder. Prevalence was also high for diagnoses of schizophrenia at 6.2% (n = 7). The mean time spent in the "Pöschwies" penitentiary at the time of the investigation was 55 months (SD = 34.98, range: 0.6–169). In nearly half of the sample (47.8%, n = 54) the index offense was murder or manslaughter. 9.8% (n = 22) of the offenders were convicted of rape, 10.6% (n = 12) of child abuse, 8.9% (n = 10) of armed robbery, 5.3% (n = 6) of physical assault, and 5.3% (n = 6) of arson. Three cases (2.7%) matched none of these categories.

### Procedure and measures

The scoring of the PCL-R as well as the assessment of psychiatric, psychological, criminological, and socio-economic variables were performed based on file data. At no time was there any direct contact with the prisoners. The files contained extensive historical details on the subject, including criminal and medical/psychiatric history, exact type and circumstances of the offense, as well as a personality assessment. To assess psychopathy, no direct contact is necessary if enough collateral information can be gathered from files and expert psychiatric assessments [2]. The interrater reliability (of n = 10 cases) was assessed with Krippendorff's alpha [33]. The advantage of this coefficient is that it can be used to analyze the agreement of multiple raters, even if there are unequal sample sizes or missing data. Furthermore, it can be computed when the variables are nominal, ordinal, or continuous. For the PCL-R, Krippendorff's alpha was 0.89.

In a first step PCL-R scores and the outcome variable were assessed. To prevent any bias, the PCL-R was scored before evaluating the outcome variable (physical and verbal aggression). Inmate behavior was assessed using the penitentiary's files. Physical aggression was defined as physical behavior that harmed or had the potential to harm others (staff members or other prisoners). Verbal aggression was defined as threats or gross verbal abuse. Damage to property was not considered an aggressive infraction. Further categories of infractions were illegal drug abuse and the possession of illegal drugs. In a second step, two psychologists coded the motives of the violent infractions, as well as their consequences, independently of each other. The motives were categorized as: (1) conflict with prison officer (threatening), (2) minor assault without physical harm, (3) starting a fight following a verbal conflict (reactive violence), (4) deliberate use of violence without prior verbal conflict (instrumental violence). The consequences of the violence used were categorized as (1) no harm, (2) minor harm, (3) moderate harm (outpatient medical treatment was necessary), and (4) severe harm (inpatient medical treatment was necessary). The interrater agreement was nearly perfect. Only in two instances the raters disagreed. After consulting the files, the raters were able to reach a consensus.

### Hypothesis and statistical analysis

The authors hypothesized that the PCL-R would be a good instrument for predicting in-prison aggressive behavior. This assumption was tested by logistic regression analyses, where physical and verbal aggression were analyzed as function of the PCL-R sum score, employing a 5% level of significance. Additionally, stratified analyses were conducted for Factor 1 and 2 of the PCL-R.

The results of all logistic regression analyses were controlled for time in prison by entering the natural log of time at risk, with its parameter fixed at 1 as a covariate, into the model.

Predictive validity was estimated with ROC analyses. All models were computed with STATA SE 10.0.

## Results and discussion

### Disciplinary infractions and classification of aggressive behavior

83.2% (n = 94) of the inmates had been reported at least once for a disciplinary infraction during incarceration. The average number of incidents per prisoner was 5.1 (SD = 5.94, range: 0–26). With regard to the type of disciplinary infraction, 13.3% (n = 15) of inmates had been reported at least once for the use or possession of illegal drugs. 25.6% (n = 29) of offenders had been reported due to at least one verbally aggressive incident, and one third of the subjects (27.4%, n = 31) had been reported at least once due to physical aggression. 79.6% (n = 90) had been reported at least once for another non-violent infraction.

For 28 of the 31 offenders behaving physically aggressive, the cause and consequences of the conflict were documented and allowed categorization: One of the offenders had a conflict with a prison officer, 7 committed a minor assault without physical harm, 17 started a fight following a verbal conflict (= reactive violence) and only 3 prisoners were deliberately violent without prior verbal conflict (= instrumental violence). Two thirds of the offenders behaving violently caused no physical harm (n = 21). Outpatient medical treatment was necessary due to 3 and inpatient medical treatment due to 4 offenders behaving violently.

### Differences between offenders with and without physically aggressive behavior

Stratified analyses with bivariate logistic regression showed no significant difference between physically aggressive and physically non-aggressive inmates with respect to marital status (p ≤ 0.84), criminal record (p ≤ 0.23), index offense (p ≤ 0.65), age at the beginning of incarceration (p ≤ 0.46), time spent in the institution (p ≤ 0.07), and vocational education (p ≤ 0.75).

### PCL-R-scores in relation to physical and verbal aggression

The PCL-R sum scores were not normally distributed. Both the mean score and the median of the PCL-R were 12 points (SD = 6.6), with scores ranging between 0 and 33.7, the 25^th ^percentile was at 6.3 points and the 75^th ^percentile at 17 points. Both the relationship between physical aggression and the sum score of the PCL-R, and the relationship between physical aggression and either of the two factors of the PCL-R, were not significant. However, both the sum score and Factor 1 predicted the occurrence of verbal aggression (AUC = 0.70 and 0.69), while Factor 2 did not (Table 1).

**Table 1:** Predictive validity of the PCL-R – Bivariate logistic regression analyses controlled for time of imprisonment

	PCL-R	OR	95% CI	AUC
PA	Sum Score	1.026	0.963–1.094	0.613
VA		*1.083	1.011–1.160	0.704
PA	Factor 1	1.040	0.911–1.188	0.610
VA		*1.158	1.007–1.331	0.686
PA	Factor 2	1.032	0.920–1.158	0.614
VA		1.075	0.955–1.210	0.672

Note:

PA = physical aggression

VA = verbal aggression, threats

AUC = Area under the curve. SE = Standard error. CI = Confidence interval. OR = Odds Ratio

*p < .05.

## Conclusion

In this study only a moderate association between PCL-R scores and prison misconduct was observed. This finding corroborates the results of several other studies [15,20,23,24,34]. More specifically, in our study the PCL-R was primarily predictive for verbal aggression (e.g. threats) but not for physical aggression (e.g. physical assaults). This finding replicates the results of the study by Edens et al. [19] and Buffington-Vollum et al. [30], where high PCL-R scores were found to be related to behavioral problems (e.g. verbal aggression) but not to physical violence.

Therefore, the question arises, how the weak relationship between PCL-R scores and physically aggressive inmate behavior can be explained. Some authors have discussed whether the low base-rate of violent infractions can be considered an explanation for the non-significant relation between PCL-R-score and violence [15]. The prevalence of violence in our study, with 27%, was reasonably high. However, the base rate of severe violence was low, as is documented by the fact that medical treatment was only necessary in 7 of the 94 cases where offenders were reported for disciplinary infractions. Low prevalence effects can therefore not be excluded from this study as a possible explanation for the weak relationship between the PCL-R score and physically aggressive behavior. It can be argued, on the other hand, that the base rate of severe violence may well have been higher, given that in most cases of violent behavior prison staff will intervene before severe violence can develop. Future investigations in the field should assess the prison staff's influence on the severity of violent inmate behavior.

There is another explanation aside from the low prevalence effect to be considered. When studying the association between psychopathic traits and violence, there is evidence suggesting that the motives of the perpetrators are important: Porter and Woodworth [35] differentiated between reactive and instrumental motivations. According to Woodworth and Porter [36] most of the violence committed by psychopaths is instrumental. Even though the base rate for violence was not low in our sample, the base rate for instrumental violence was very low. Most of the violent infractions in the Zurich state penitentiary were reactive in nature as they resulted from verbal disagreements which led to marginal or moderate physical violence. Since the inmates examined in our study did not display an instrumental type of violence, our findings do not rule out the usefulness of the PCL-R to predict intramural violence. Furthermore, it has to be considered that in our sample the mean PCL-R score, with 12 points, was low – even though it was comparable to other studies from German speaking countries [37]. Moreover, according to Buffington-Vollum et al. [30], restrictive environmental factors may inhibit the aggressive tendencies of people who might be violent in less restrictive settings. The Zurich state penitentiary is a highly controlled environment that is designed to prevent violence: The inmates have single cells – this measure increases the costs but allows privacy, which in turn lowers the prevalence of violent behavior through the seclusion of inmates for 12 hours a day. A workday is typically spent in small groups constantly supervised by staff members, who immediately respond to violence (verbal or physical) by separating the perpetrators and reporting them to the prison directorate. Furthermore, there are nine psychologists, two psychiatrists, and two general practitioners taking care of 316 inmates (of the maximum security unit). The ratio of mental health professionals to inmates of 1:24 can be considered high and could also be a relevant factor in reducing in-prison violence. In addition, many offenders receive offense-oriented forensic psychotherapy during imprisonment, which aims at reducing aggressive tendencies and trains empathy in offenders, both of which may help lower prevalence of aggressive incidences.

Since most of the intramural violence observed could be classified as reactive, the usefulness of the PCL-R – especially when using only the sum score as predictor – is questionable – especially in highly controlled prison settings such as the Zurich state penitentiary. Another recent study from Switzerland showed that the Violence Risk-Appraisal Guide (VRAG) [38] also failed to predict violent infractions [39], indicating that not only the PCL-R is problematic when it comes to predicting in-prison violence. Thus the development of a specific instrument or model to predict *reactive *institutional violence seems to be needed. Furthermore, future research should assess specifically, whether the PCL-R is useful to predict *instrumental *in-prison violence, rather than trying to assess if it can predict in-prison violence in general.

## Competing interests

The authors declare that they have no competing interests.

## Authors' contributions

JE has made substantial contributions to the conception and design of the study, as well as the acquisition, analysis and interpretation of data and drafting of the manuscript. He has given his final approval for this version of the manuscript to be published. FU has made substantial contributions to the analysis and interpretation of data and has been involved in critically revising the manuscript. He has given his final approval for this version of the manuscript to be published. AL has made substantial contributions to the analysis and interpretation of data, has been involved in drafting and critically revising the manuscript. She has given her final approval for this version of the manuscript to be published. AR has made substantial contributions to the conception and design of the study, as well as the acquisition, analysis and interpretation of data and been involved in drafting the manuscript. She has given her final approval for this version of the manuscript to be published. SV has made substantial contributions to the conception and design of this study and has been involved in critically drafting and revising the manuscript. He has given his final approval for this version of the manuscript to be published.

## Pre-publication history

The pre-publication history for this paper can be accessed here:


